# The Effect of Smart PEO-Coatings Impregnated with Corrosion Inhibitors on the Protective Properties of AlMg3 Aluminum Alloy

**DOI:** 10.3390/ma16062215

**Published:** 2023-03-09

**Authors:** Andrey S. Gnedenkov, Yana I. Kononenko, Sergey L. Sinebryukhov, Valeriia S. Filonina, Igor E. Vyaliy, Alexey D. Nomerovskii, Alexander Yu. Ustinov, Sergey V. Gnedenkov

**Affiliations:** Institute of Chemistry, Far Eastern Branch of the Russian Academy of Sciences, 159 Pr. 100-Letiya Vladivostoka, 690022 Vladivostok, Russia; asg17@mail.com (A.S.G.);

**Keywords:** aluminum, aluminum alloys, corrosion protection, plasma electrolytic oxidation (PEO), corrosion inhibitors, benzotriazole, 1,2,4-triazole, polyvinilidene fluoride (PVDF)

## Abstract

The protective coating with a self-organized microtubular structure was formed using plasma electrolytic oxidation (PEO) on AlMg3 aluminum alloy in the tartrate-fluoride electrolyte. This protective layer was further modified using corrosion inhibitors of the azole group (1,2,4-triazole, benzotriazole) and polymer material (polyvinilidene fluoride, PVDF). X-ray diffraction analysis and scanning electron microscopy with energy dispersive spectroscopy were used to study the morphology and composition of the obtained oxide coatings. The presence of the inhibitor in the PEO-layer was confirmed using micro-Raman spectroscopy and X-ray photoelectron spectroscopy. The level of corrosion protection of formed coatings as well as the effect of loaded inhibitors on the anticorrosion efficiency was evaluated using electrochemical impedance spectroscopy (EIS) and localized scanning techniques (SVET/SIET). The coating impregnation with corrosion inhibitors of the azole group significantly improves the corrosion characteristics of the material. Impregnation of the base PEO-layer with 1,2,4-triazole during 24 h results in a 36 times increase in the impedance modulus measured at the lowest frequency (|Z|*_f_*_=0.1Hz_). Additional sealing of impregnated coating with polymer improves the corrosion stability of the treated material. On the base of the obtained data, the optimal way of protective inhibitor- and polymer-containing formation using surface treatment was suggested. The best barrier properties were established for hybrid coatings obtained by the immersion of a PEO-coated sample in 1,2,4-triazole solution for 24 h and following spraying the PVDF solution. The value of |Z|*_f_*_=0.1Hz_ for this protective layer increased by more than two orders of magnitude in comparison with the base PEO-layer. The three-stage mechanism of corrosion inhibition of the sample with smart inhibitor-containing coating was established.

## 1. Introduction

Aluminum and its alloys, due to their light weight, strength, and ductility, hold a leading position among non-ferrous metals in terms of the scale of use in production and industry [1]. Aluminum alloys are characterized by high impact strength, due to which they have become one of the most important structural materials in the aircraft industry [2]. However, the operation of metal products in aggressive environments (sea water, salt fog, high humidity, etc.) leads to their corrosion and resource limitation [3,4]. Corrosion processes of metals are directly related to safety and economic efficiency. As soon as corrosion occurs, the mechanical properties of the material begin to deteriorate rapidly, and if the corrosion centers are not eliminated in time, it is often necessary to replace the entire product with the new one. The development of technologies focused on the increase of metals’ corrosion resistance is an urgent task today [5,6,7,8].

In the last few decades, priority has been given to coating technologies [9,10,11,12]. The advantage of these methods is not only the possibility of increasing the corrosion resistance of the metal, but also increasing the hardness, wear, and heat resistance, and the possibility of modifying the electrical properties (electrical insulating or electrically conductive). Coatings obtained using plasma electrolytic oxidation (PEO) have excellent adhesion to a substrate (including aluminum and its alloys) [13]. These coatings are also characterized by ease of formation and a wide range of composition, microstructure, porosity, and roughness of the resulting layers, which was provided by varying the composition of the electrolyte and modes of the PEO process [14,15,16,17]. Many research groups used the PEO method to modify the surface of the aluminum alloys. Truong et al. [3] formed PEO-layer on D16T aluminum alloy in an alkali-silicate electrolyte with the addition of Co(OH)_2_, which decreases the corrosion rate up to 24 times. Lee et al. [8] showed that the addition of the carbon nanotube in the silicate electrolyte for PEO promoted the decrease in the size of micropores. Xue et al. [4] used the PEO method to obtain a corrosion-resistant ceramic film on 2024 Al alloy/SiC composite in silicate solution. Zhu et al. [5] applied the PEO-layer to increase the corrosion resistance of the 7075 Al alloy and studied the effect of polarization frequency on microstructure, chemical composition, and protective properties of the formed coating. Dai et al. [1] studied the effect of surface substrate roughness on the fatigue life of PEO-coated 2024-T3 Al alloy. Wang et al. [6] showed that the addition of Ce(SO_4_)_2_ in silicate-hydroxide electrolytes improved the homogeneity and corrosion resistance of the PEO-coatings formed on ZL108 Al alloys. Zhu et al. [18] established that the increase in the negative current during the PEO process promoted the improvement of the compactness and corrosion resistance of the coating formed on 7075 Al alloy in the multicomponent electrolyte. Nadimi et al. [19] indicated that the addition of SIO_2_ nanoparticles into the composition of phosphate-hydroxide electrolyte supported the corrosion and wear resistance of the base PEO-layer. The PEO-coating was also formed to increase the corrosion and wear performance of the cold-sprayed 7075 aluminum alloy [20]. All these studies confirmed the high efficiency of the PEO method in improving the surface properties of Al-based materials.

Unfortunately, in some cases, due to PEO-coatings morphology, the level of their protective properties may be insufficient. However, the high porosity of the PEO-layer makes it possible to use these coatings as containers for loading with different modifying agents, such as corrosion inhibitors or polymers, to increase the material’s corrosion resistance. Lu et al. [21,22] modified the PEO-layer formed on 2024 Al alloy in silicate electrolyte using the treatment in PTFE solution. Pezatto et al. [23] used PEO-coating on 7020 AA and AZ80 alloys as a pre-treatment for sol-gel formation. The authors in the recent review summarized the studies that dealt with the formation, features, and application of the PEO-coating modified with different types of polymers [24]. As a result of the abovementioned surface treatment, composite coatings (CC) with two-factor protection were obtained. The formation of such coatings, which reduce the corrosion rate of metals and alloys, has been widely studied in the last decade [24,25,26].

One of the most common ways to protect metals against aggressive environments is corrosion inhibition. There are numerous works, which show that substances of inorganic and organic nature can act as inhibitory agents: chromates, nitrites, vanadates, molybdates, natural gums, amines, quinolines, imidazole and pyridine derivatives, phthalocyanines, biopolymers/hydrocarbon polymers, etc. [27,28,29,30,31,32,33,34,35,36,37,38,39,40,41,42,43,44,45,46,47,48]. Among them, substances from the azole group (1,2,4-triazole, benzotriazole, etc.), which reduce the rate of both anodic and cathodic processes, are highly effective in inhibiting aluminum corrosion [49,50]. Moreover, inhibitors of this group are non-hazardous (in comparison with many inorganic corrosion inhibitors) and accessible (as compared to inhibitors containing rare earth elements).

The objective of this research was to develop the method of formation of protective triazole-containing coatings with the prolonged level of corrosion protection of AlMg3 aluminum alloy. The PEO-coating impregnation with inhibitor (benzotriazole and 1,2,4-triazole) was intended to supply the oxide layer with self-healing properties, which provide the active corrosion protection of the material. In the presented study, the proper matrix for impregnation was formed on the base of PEO-coating with a microtubular structure. To increase the level of anti-corrosion properties of the formed inhibitor-containing composite coatings, the treatment with polymer material–polyvinylidene fluoride (PVDF) was performed. Scanning electron microscopy (SEM)/energy dispersive X-ray spectrometry (EDS), X-ray diffraction (XRD), confocal micro-Raman spectroscopy, photoelectron spectroscopy (XPS), and scratch testing were complementarily employed to study the chemical/phase composition, structure, morphology, and adhesion of the formed protective layers on Al alloy surface. The system protection was verified using electrochemical impedance spectroscopy (EIS) and localized scanning electrochemical methods: SVET (scanning vibrating electrode technique) and SIET (scanning ion-selective electrode technique). The corrosion mechanism of coated Al alloy in chloride-containing solutions was established, which is beneficial for better understanding the degradation behavior of the promising constructional material. The results indicate that the application of fluoropolymer material in combination with active corrosion inhibitors significantly increases the corrosion resistance as well as the uniformity of the surface topography of coatings as compared to the base PEO-layer. According to the results of the literature data analysis, such studies haven’t been carried out previously by research teams to the best of our knowledge.

## 2. Materials and Methods

### 2.1. Sample Characterization and Processing

Rectangular sheets made from aluminum-magnesium alloy AlMg3 (wt.%: Mg—3.8; Mn—0.6; Si—0.8; Fe—up to 0.5; Ti, Cu, Zn—up to 0.1 each, Al—balance) were used in this study as a substrate. The size of the samples was 35 × 45 × 2 mm. Before coating, the specimens were ground on a TwinPrep 5x grinding/polishing machine (Allied High Tech Products, Inc., Compton, CA, USA). Grinding was performed using silicon-carbide paper with a gradual reduction in the abrasive grain size down to 14–20 µm (P1000). After wet grinding, the samples were degreased with ethanol and dried in air.

### 2.2. PEO-Coating Formation

PEO-coatings on AlMg3 samples were formed in the galvanostatic mode at the current density of 0.95 A∙cm^−2^. The duration of the oxidation time was 40 s. The electrolyte was an aqueous solution of sodium fluoride (NaF, 35 g/L) and potassium tartrate (K_2_C_4_H_4_O_6_, 0.6 g/L).

### 2.3. Inhibitor and Polymer Treatment

PEO-coated samples were impregnated with corrosion inhibitors 1,2,4-triazole or benzotriazole from a 0.05 M aqueous solution. This concentration of the inhibitor is the optimal one and is widely used by various scientific groups [51,52,53]. The pH of both inhibitor-containing solutions for impregnation of the PEO-coated sample was neutralized (pH = 7.0–7.2). To ensure the best filling of tubular micro-containers of the PEO-layer with corrosion inhibitors, the impregnation was carried out under a vacuum in an Epovac vacuum impregnation apparatus (Struers, Copenhagen, Denmark). After vacuum treatment, the samples were immersed in the solution under constant steering for 0.5, 1, 2, and 24 h to determine the most optimal impregnation method. Then, the samples were slowly withdrawn from the solution and dried in a desiccator at 40 °C for 24 h.

The samples that showed the best anti-corrosion characteristics were treated with polyvinilidene fluoride (PVDF) to enhance the protective properties of the formed composite coatings (CC). The polymer was dissolved in methylpyrrolidone in a ratio of 1:20 and applied by spray-coating method onto the surface of samples with composite inhibitor-containing coating. The thickness of the PVDF-layer was 3 µm. The inhibitor- and polymer-containing coatings are classified as hybrid coatings (HC). As a result of the described techniques, the following types of coated samples were obtained:

PEO–sample with base PEO-layer.
CC-1,2,4-tr-0.5, CC-1,2,4-tr-1, CC-1,2,4-tr-2, CC-1,2,4-tr-24–samples with composite coating, obtained by the immersion of the PEO-coated specimen in 1,2,4-triazole solution for 0.5 h, 1 h, 2 h, and 24 h, respectively.
CC-btr-0.5, CC-btr-1, CC-btr-2, CC-btr-24–samples with composite coating, obtained by the immersion of the PEO-coated specimen in benzotriazole solution for 0.5 h, 1 h, 2 h, and 24 h, respectively.
CC-P–sample with composite coating, obtained by the treating the PEO-coated specimen with PVDF using spray-coating method.
HC-1,2,4-tr, HC-btr–samples with hybrid coating obtained by treatment of CC-1,2,4tr-24 and CC-btr-24 specimens, respectively, with PVDF using spray-coating method.

**Figure 1:**
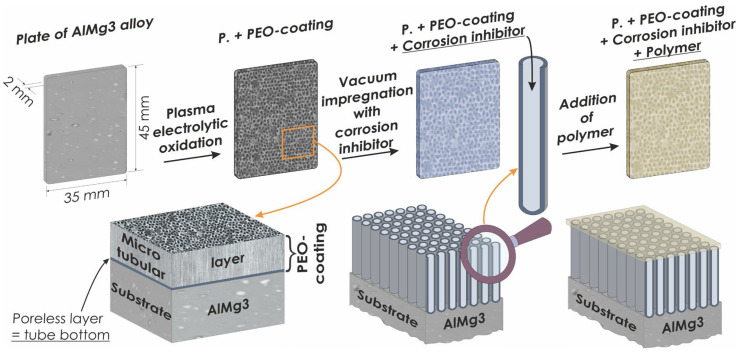
Stages of formation of composite and hybrid coatings.

The scheme describing the formation of composite and hybrid coatings on AlMg3 aluminum alloy is shown in Figure 1.

### 2.4. Coatings’ Characterization

#### 2.4.1. SEM-EDS Analysis

Scanning electron microscopy (SEM) and energy dispersive spectroscopy (EDS) were used to determine the structure of the PEO-layer and its elemental composition. An investigation was carried out using an EVO 40 (Carl Zeiss, Munchen, Germany) scanning electron microscope equipped with Silicon Drift Detector X-MaxN 80 (Oxford Instruments NanoAnalysis, Concord, MA, USA).

For cross-section preparation, the specimens were fixed in epoxy resin (EpoxySet#145-20005, Allied High Tech Products Inc., USA). The FixiForm casting mold (Struers A/S, Ballerup, Denmark) with a 30 mm diameter was used. Samples were ground and polished using a Tegramin-25 machine (Struers A/S, Ballerup, Denmark). For this process, the sample was treated by means of fine grinding papers (SiC Foil, MD-Largo, Struers A/S, Ballerup, Denmark) and polishing discs (MD-Mol, MD-Chem, Struers A/S, Ballerup, Denmark). The diamond suspensions (DP-Suspension P, Struers A/S, Copenhagen, Denmark) with an abrasive size in the range of 9–3 µm together with DP-Lubricant Brown were used.

#### 2.4.2. Raman Spectroscopy

Confocal micro-Raman spectroscopy was used to confirm the impregnation of the PEO-coating with corrosion inhibitor of the triazole group: benzotriazole and 1,2,4-triazole (CC-btr-24 and CC-1,2,4-tr-24 samples). Raman scattering spectra were acquired in the range from 200 to 1300 cm^−1^ (for a CC-btr-24 sample) and 800 to 1600 cm^−1^ (for a CC-1,2,4-tr-24 sample) using confocal Alpha 500 spectrometer (WITec, Ulm, Germany) and WITec Control/Project Plus 2.1 software. The Raman spectra were recorded using a green laser (wavelength 532 nm) with a radiation power of 30 mV for 10 min (60 accumulated spectra). The distribution of benzotriazole and 1,2,4-triazole over the surface of the PEO-layer was presented as 2D intensity maps. The maps were built as a result of scanning a selected coverage area with the size of 25 × 25 μm containing 50 × 50 spectra. In the scanning mode, the integration time for spectrum recording was 1 s.

#### 2.4.3. XRD Analysis

X-ray diffraction (XRD) analysis of specimens with PEO-layers was performed by means of a SmartLab diffractometer (Rigaku, Tokyo, Japan). CuK_β_ radiation and Bragg–Brentano geometry was used for XRD analysis. The range of spectrum acquiring was 2θ = 4–90°. Spectrum was obtained with a step of 0.01°, a generator current of 140 mA, and a voltage of 42 kV at room temperature.

#### 2.4.4. XPS Analysis

The chemical analysis of CC-1,2,4-tr-24 and CC-btr-24 obtained on a AlMg3 aluminum alloy was performed using X-ray photoelectron spectroscopy (XPS). The surface was studied under ultrahigh vacuum (0.5 µPa) by means of a spectrometric complex SPECS (SPECS GmbH, Berlin, Germany) with a hemispherical electrostatic energy analyzer PHOIBOS-150 using non-monochromatic AlKα radiation with an energy of 1486.6 eV. The aliphatic carbon line (C1s line) with a binding energy of 285.0 eV was used for spectra calibration. The surface of specimens was Ar^+^-etched for 3 min at an energy of 5000 eV to remove the contaminators and to provide the analysis of the inner layers of the formed coating. The ion etching results in coating thickness decreasing by 3–5 nm.

### 2.5. Electrochemical Research

#### 2.5.1. Electrochemical Impedance Spectroscopy

The level of protective properties of the obtained coatings was evaluated by electrochemical impedance spectroscopy (EIS). The studies were carried out using a 12558WB system (Solartron Analytical, Hampshire, Great Britain) consisting of an SI 1287 electrochemical interface and an FRA 1255B frequency response analyzer. The measurements were carried out in a three-electrode cell Model K0235 Flat (PAR, Oak Ridge, TN, USA) with the saturated silver chloride (Ag/AgCl) electrode (the potential versus standard hydrogen electrode is equal to 0.197 V) as a reference electrode and the platinized niobium mesh as a counter electrode. The 3.5 wt.% sodium chloride solution was used as an electrolyte. The sample area exposed to electrolyte was equal to 1 cm^2^. Before impedance measurements, the sample was kept in the electrolyte for 60 min to achieve the stabilization of the electrode potential. Registration of impedance spectra was carried out in the frequency range from 1 MHz to 0.1 Hz with a logarithmic sweep of 10 points per decade. The EIS measurements were carried out during 24 h sample exposition to the electrolyte. The impedance spectra were recorded every 2 h.

#### 2.5.2. SVET/SIET Studies

The local corrosion behavior of studied samples was estimated using local scanning electrochemical techniques. The SVET (scanning vibrating electrode technique) allows for the detection of the changes of local current density occurring due to the difference of Galvani potentials in microgalavanic cells formed as a result of the heterogeneity of the materials phase composition. Determination of local ionic concentration shift (H^+^, Cl^−^, Mg^2+^, Na^+^, etc.) in solution near the sample’s surface is performed by SIET (scanning ion-selective technique).

SVET measurements were performed using a Pt-Ir wire. A Pt black was deposited on an electrode’s spherical tip with a diameter of 10 µm. Probe vibrations were implemented in X and Z axis (horizontal and vertical directions, respectively, relative to the studied surface) with a frequency of 128 Hz (X) and 325 Hz (Z). The amplitude was 25 µm. Vertical vibration (Z axis) was used to calculate the corrosion current density.

SIET (scanning ion-selective electrode technique) studies were carried out using a microelectrode consisting of a glass capillary (an outer diameter–1.5 mm, conical tip diameter–2.0 ± 0.5 µm) and a silver wire coated with silver chloride (Ag/AgCl) as an inner reference electrode. The capillary tip was filled with hydrogen-selective (H^+^) membrane Hydrogen ionophore II–cocktail A from Selectophore, Honeywell Fluka, USA (column length was 60–70 µm), and the rest of the capillary space was filled with the inner reference solution (0.01 M KH_2_PO_4_ in 0.1 M KCl). The scanning ion-selective electrode was located at 40 ± 5 µm from the sample’s surface. To avoid possible mixing of the electrolyte and failure of the glass SIET electrode due to multidirectional vibrations of the SVET probe, its position relative to the ion-selective microelectrode was described by the coordinates 40 µm, 20 µm, and 40 µm along the X, Y, and Z axes, respectively. The drift of the potential during the test was measured using a Sentron-SI pH-meter with a MiniFET electrode (pH-electrode, #9202-010, SENTRON Europe B.V., Roden, The Netherlands). In addition to the installed SVET/SIET microelectrodes, the system includes an external silver chloride (Ag/AgCl) reference electrode.

Experiments were carried out in a 0.05 M NaCl solution due to the best signal/noise ratio. Calibration of a SIET electrode was realized according to the Nernst equation using buffer solutions. The Nernst slope was 57 ± 0.6 mV∙pH^−1^. Local electrochemical measurements were controlled using ASET 2.0 software (ScienceWares, Falmouth, MA, USA). A preamplifier with 1 PΩ input impedance was used to measure the potential. Quasi-simultaneous SVET/SIET measurements were made on AlMg3 aluminum alloy PEO-coating, CC-btr-24, and CC-1,2,4-tr-24 samples.

### 2.6. Scratch Testing

The adhesion of the inhibitor-containing PEO-coating treated with PVDF was studied using the scratch test. Measurements were performed using the Revetest Scratch Tester (CSM Instruments, Peseux, Switzerland) by means of Rockwell diamond indenter. The increase of the load was from 1 up to 100 N at a rate of 12 N min^−1^. The track length was about 5 mm.

## 3. Results and Discussion

### 3.1. Characterization of the Protective Layer

#### 3.1.1. Morphology and Elemental Analysis (SEM-EDS Data)

**Figure 2:**
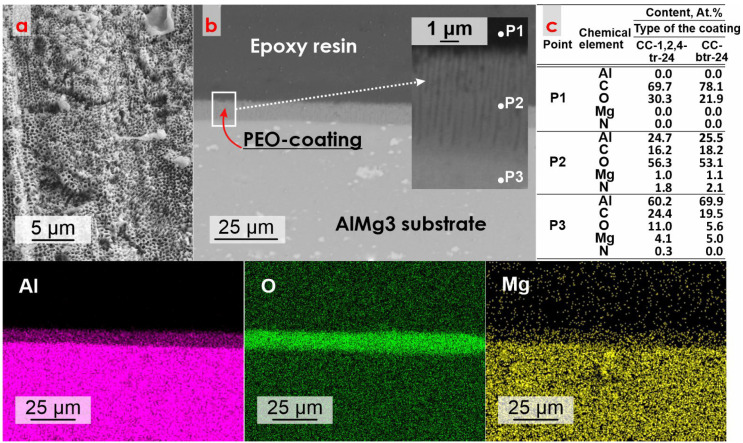
SEM/EDS data of the morphology (**a**,**b**) and elemental composition of the PEO-coating. EDS data of the element distribution at three points in the cross-section of CC-1,2,4-tr-24 and CC-btr-24 (**c**). The arrow shows the place at composite coating (PEO-layer presented as an example), where EDS spectra were acquired.

Figure 2 presents the data on the morphology and composition of the obtained coating, detected by SEM/EDS analysis. According to the analysis of the presented data, it can be concluded that the PEO-coating has a microporous structure. The average pore diameter varies from 200 to 300 nm (Figure 2a). Analysis of the SEM-image of the sample’s cross-section allowed to reveal that formed microtubes have an ordered self-organized structure with the average tube height equaled to ~10 µm (Figure 2b). Based on the data of PEO-coating’s morphology, it can be assumed that formed surface structures can serve as microcontainers for further impregnation with corrosion inhibitors. From the analysis of EDS data, it was concluded that the PEO-coating composition includes aluminum and oxygen (Figure 2). Magnesium was also found since it is the main alloying element in the AlMg3 alloy. To confirm the presence of corrosion inhibitors in the pores of composite PEO-coatings for CC-btr-24 and CC-1,2,4,-tr-24 samples, the EDS spectra were recorded in three different points (P1, P2, P3) of sample cross sections (P1 is epoxy resin, P2 is CC-coating, and P3 is AlMg3 substrate). The presence of nitrogen at the P2 point of the cross-sections of samples with inhibitor-containing coatings (Figure 2c) and the absence of N at two other points prove the loading of formed micro-containers with corrosion inhibitors.

#### 3.1.2. Phase Analysis (XRD Data)

**Figure 3:**
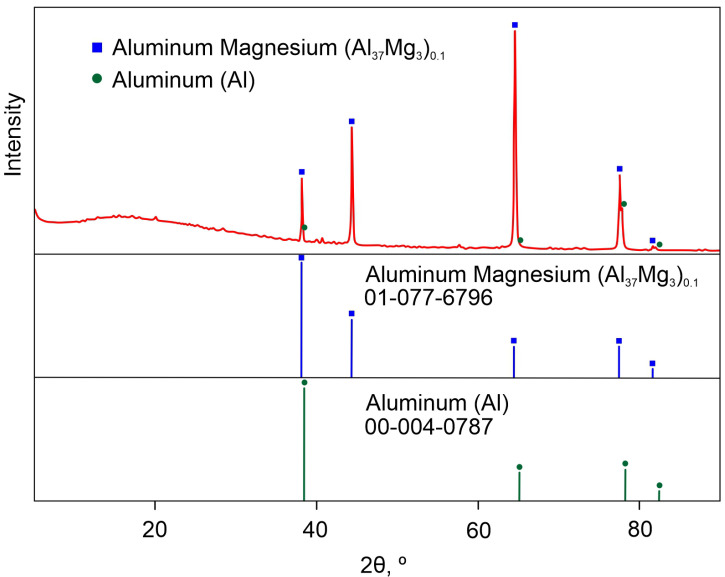
XRD pattern of PEO-coated aluminum sample.

According to the results of XRD analysis, only the aluminum (Al) and aluminum magnesium (Al_37_Mg_3_)_0_._1_ intermetallic compound belonging to the substrate were detected as the crystal phases in the composition of the PEO-coated sample (Figure 3). Al-Mg intermetalide in the composition of the substrate is a result of alloying the aluminum with magnesium [54]. Based on the presented data, it can be assumed, that Al- and O-containing compounds (probably Al_2_O_3_), spotted by the EDS method are in the amorphous state, which can be related to the wide halo shown at low angles (up to 30°). However, if we take into account the harsh conditions realized during PEO process (temperature in the plasma discharge channels up to 10^4^ K) and pressure (up to 10^2^ MPa), the presence of aluminum in metallic form and (Al_37_Mg_3_)_0_._1_ in the coating composition (according to XRD results) is impossible and was caused with low X-ray reflectance of PEO-layers. As a result of high heating and sharp cooling the sample (duration of discharge lifetime equal to 10–200 µS) the substance on the substrate/coating interphase transfers into an amorphous condition. XPS data presented below confirm this suggestion.

#### 3.1.3. Chemical Composition Analysis (XPS Data)

**Figure 4:**
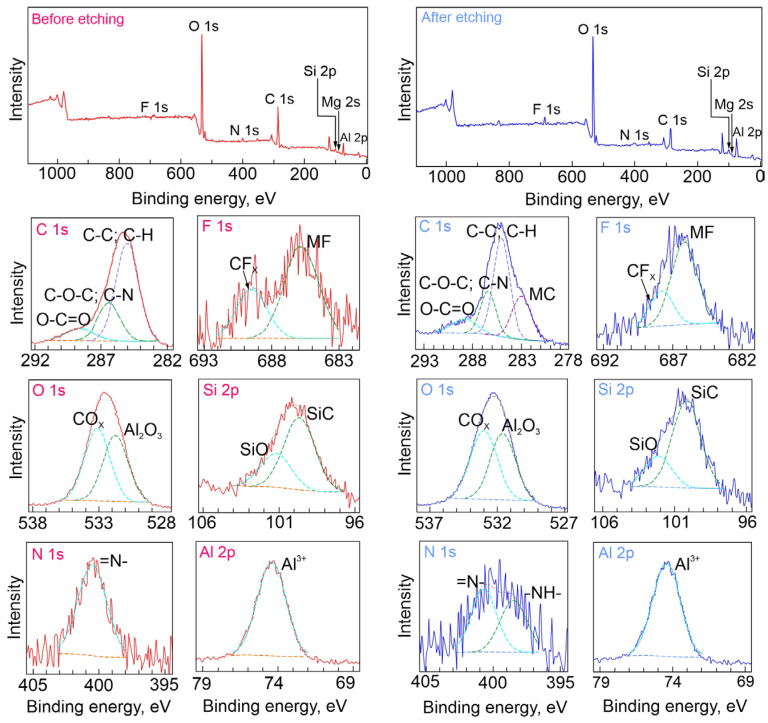
XPS spectra obtained for a sample with 1,2,4-triazole containing composite coating (0.05 M) and the detailed high resolution spectra before and after Ar^+^ etching.

**Figure 5:**
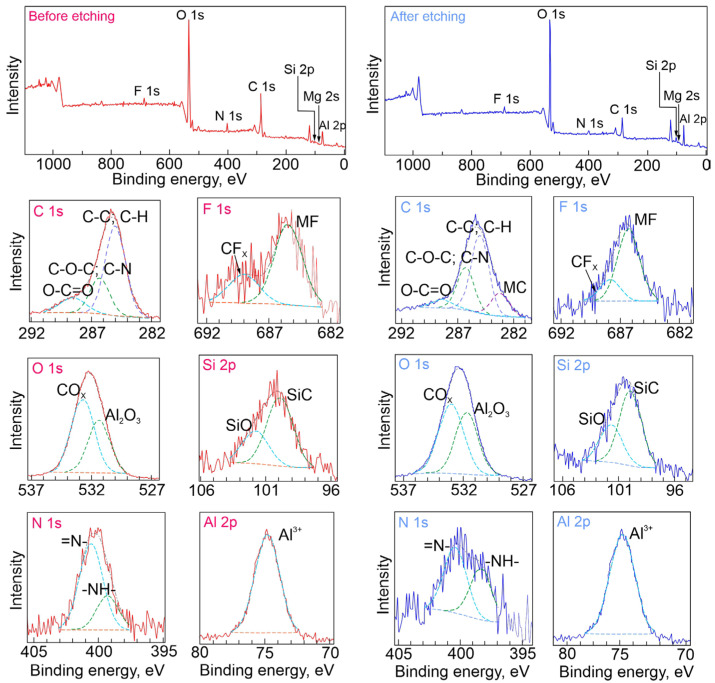
XPS spectra obtained for a sample with benzotriazole containing composite coating (0.05 M) and the detailed high resolution spectra before and after Ar^+^ etching.

X-ray photoelectron spectroscopy was used for the determination of chemical composition of the formed protective composite layers. The CC-1,2,4-tr-24 and CC-btr-24 samples were analyzed by the XPS method. The CC-1,2,4-tr-24 sample (before etching, Figure 4) contains a significant amount of oxygen, carbon, and aluminum (Table 1). The binding energy (*E_b_*) of Al 2p (74.4 eV) and O 1s (at *E_b_* = 531.7 eV) demonstrates the presence of Al_2_O_3_ in the analyzed layer. Nitrogen of both “pyrrole” and “pyridine” types, detected at binding energies 400 and 399 eV is related to the presence of these forms of the heteroatom in 1,2,4-triazole (Figure 4, Table 1) and benzotriazole (Figure 5, Table 2) compounds. After etching (3 min, *E* (Ar^+^) = 5000 eV), the proportion of aluminum noticeably increases, while that of carbon decreases. This indicates the removal of contaminants and part of the inhibitor-containing layer. Spectra obtained for a benzotriazole-containing sample CC-btr-24 (Figure 5, Table 2) are comparable to the ones observed for the sample with 1,2,4-triazole-containing coating CC-1,2,4-tr-24. The content and state of the respective chemical elements differ slightly. The similarity of the C 1s spectra for 1,2,4,-triazole and benzotriazole-containing samples indicates a similar contribution of the inhibitor agent to the composition of the analyzed layer (about 3–5 nm thick), in which PEO structures, primarily Al_2_O_3_, dominate. XPS analysis also detects such chemical states of elements in the coating composition as: CF_x_, CO_x_, MF*, MC*, SiO, SiC, Mg^2+^ (M stands for the metal), which indicates the formation of different substances as a result of PEO process. Mg and Si are the alloying elements of the AlMg3, whereas C, O, and F are related to the composition of the electrolyte. The presence of the C-N in C 1s spectra also confirms the coating impregnation with azole inhibitors.

#### 3.1.4. Chemical Composition Analysis (Micro-Raman Spectroscopy Data)

Confocal micro-Raman spectroscopy was used to determine the chemical composition of the CC-1,2,4-tr-24 and CC-btr-24 samples. This method, in the scanning mode of the surface area under study, also makes it possible to determine at the microscale the distribution of one of the components that make up the protective layer. To confirm the impregnation of benzotriazole and 1,2,4-triazole into the structure of the PEO-coating, images of the studied area of the sample, distribution maps of benzotriazole and 1,2,4-triazole were obtained. The following spectra were also obtained: Raman spectra of powders of benzotriazole and 1,2,4-triazole and Raman spectra of surface areas corresponding to areas with a high and low concentrations of inhibitor in the coating.

**Figure 6:**
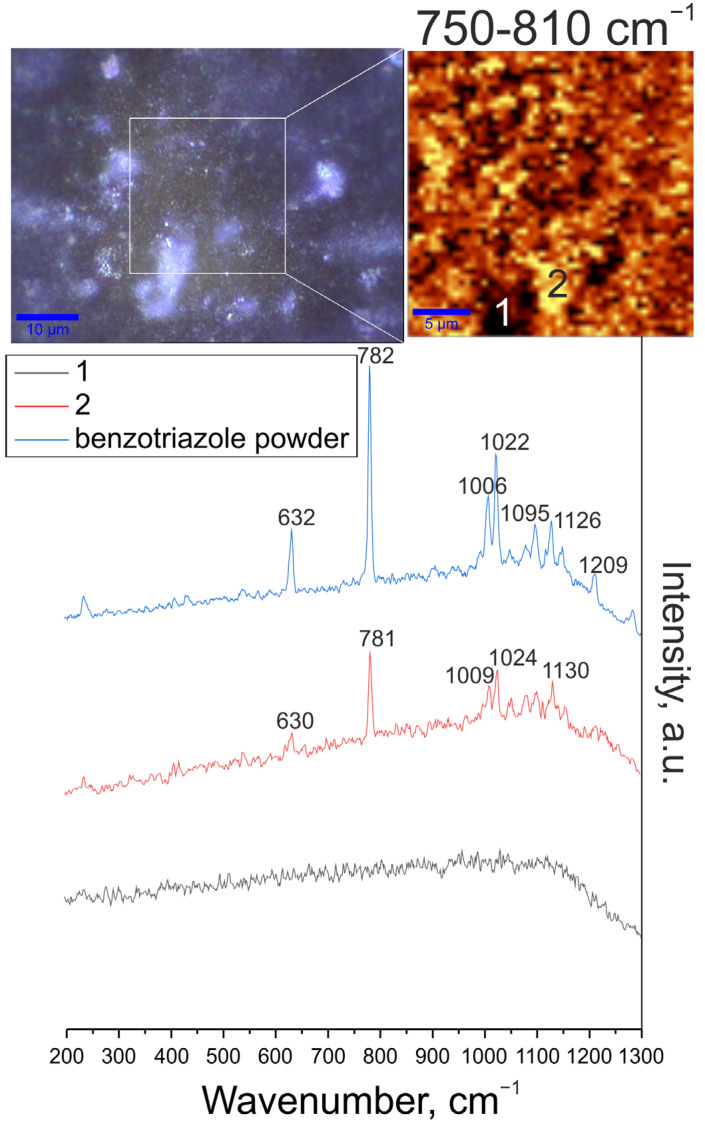
Image of the studied region in the sample, map of the benzotriazole distribution (2D map was designed using a filter in the spectral range of 750–850 cm^−1^ corresponding to the characteristic peak for benzotriazole), Raman spectrum of benzotriazole powder and Raman spectra of surface areas corresponding to areas of high and low content of the inhibitor.

Analysis of the spectrum of benzotriazole powder indicates the corresponding bands characteristic of this inhibitor (Figure 6). An intense characteristic peak at 782 cm^−1^ was fixed, which is responsible for the so-called “breathing” vibrations of the C = C bonds of the benzene ring. Peaks at 1006 cm^−1^ and 1022 cm^−1^ are associated with bending vibrations of bonds (caused by stretching of C = C skeletal bonds in the benzene ring, as well as δ(CH)) [55,56]. Peaks at 1095 cm^−1^ and 1126 cm^−1^ refer to bending vibrations δ(NH) and δ(CH), respectively. The narrow peak at 1209 cm^−1^ is responsible for the combination of asymmetric stretching vibrations ν(N–N–N) and bending vibrations δ(NH). The peak at 632 cm^−1^ describes the torsional vibrations of bonds in the triazole ring [55,56,57].

**Figure 7:**
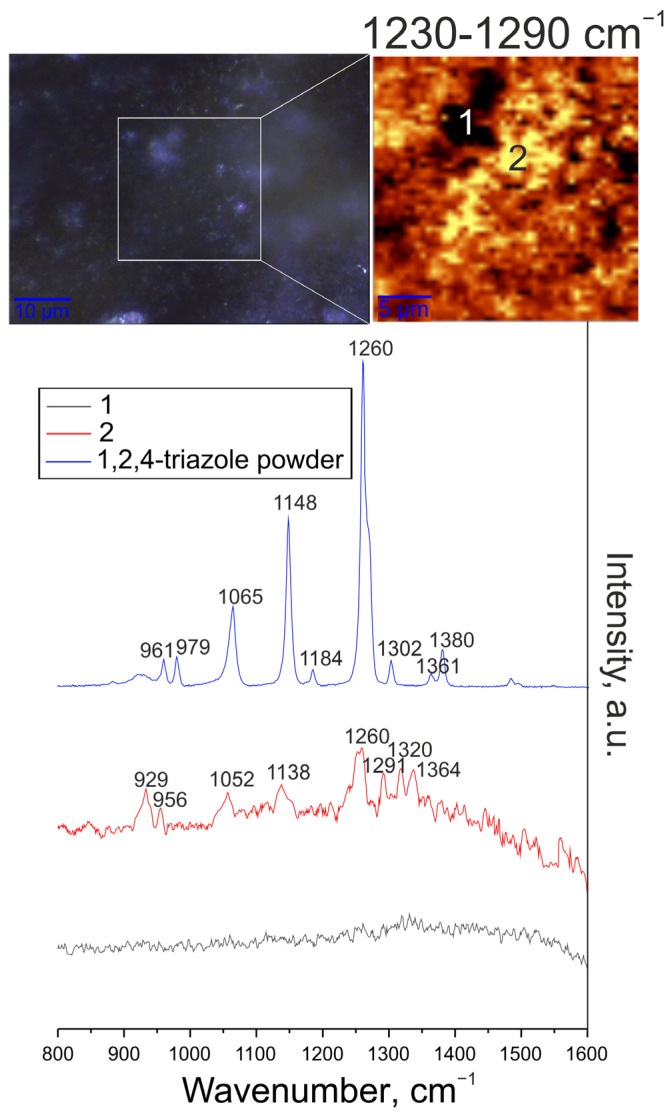
Image of the studied region in the sample, map of the 1,2,4-triazole distribution (2D map was designed using a filter in the spectral range of 1200–1300 cm^−1^ corresponding to the characteristic peak for 1,2,4-triazole), Raman spectrum of 1,2,4-triazole powder and Raman spectra of surface areas corresponding to areas of high and low content of the inhibitor.

Figure 7 shows the image of the studied area of the sample, the distribution map of 1,2,4-triazole, the Raman spectrum of powder 1,2,4-triazole, and Raman spectra of surface areas corresponding to regions with high and low inhibitor content. Analysis of the 1,2,4-triazole spectrum indicates the presence of corresponding bands characteristic of this inhibitor. The strong characteristic peak was recorded at 1260 cm^−1^, which is responsible for the stretching vibrations of the N = N double bond of the triazole ring ν(N = N) [58]. The medium peak at 1065 cm^−1^ is responsible for the combination of in-plane bending vibrations δ(NH) and δ(CH) [58,59]. The peak at 1302 cm^−1^ is associated with in-plane bending vibrations of the δ(CH) bonds [58,60]. The strong peak at 1148 cm^−1^ refers to the stretching vibrations of the N–N single bond of the triazole ring ν(N–N) [59], which appears in the course of tautomeric transformations. Peaks at 1184 cm^−1^ and 961 cm^−1^ refer to in-plane bending vibrations of δ(NNH) and δ(NCN), respectively [58,61,62]. The peak at 1361 cm^−1^ describes the stretching of the 1,2,4-triazole ring [60], and the peak at 979 cm^−1^ describes the in-plane bending vibrations of the 1,2,4-triazole ring (δ_ring_) [58]. The bond at 1380 cm^−1^ refers to stretching vibrations ν(C–N) [58,61].

To determine the distribution intensity of benzotriazole and 1,2,4-triazole over the surface of the coated samples, 2D maps were designed using a filter in the spectral range of corresponding with most intensive characteristic peaks, namely 750–810 cm^−1^ for benzotriazole (“breathing” vibrations of C = C bonds of the benzene ring at 790 cm^−1^) and 1230–1290 cm^−1^ for 1,2,4-triazole (stretching vibrations of the N = N double bond of the triazole ring ν(N = N)). An optical image of the studied coating areas (highlighted by a frame) and a 2D map showing the distribution of benzotriazole and 1,2,4-triazole over the surface are presented in Figure 6 and Figure 7. An analysis of the experimental data indicates the distribution of the inhibitor over the coating surface. The area of light zones (areas with a high concentration of benzotriazole and 1,2,4-triazole) is significantly higher than the dark areas. The lower concentration of the inhibitor recorded in the dark areas may be due to the complex morphology of the coating and the deposition of most parts of the inhibitor in the pores of the protective layer. The spectra recorded in the regions marked by 1 and 2 correspond to the areas with high and low inhibitor content. 

The spectrum for the coating with benzotriazole clearly shows bands at 630, 781, 1009, 1024, and 1130 cm^−1^, which correspond to bond vibrations in benzotriazole (Figure 6). The spectrum for the coating with 1,2,4-triazole reveals the bands at 929, 956, 1052, 1138, 1260, 1291, 1320, and 1364 cm^−1^, which are related to bond vibrations in 1,2,4-triazole. The slight shift of the peaks relative to the pure substance is probably due to the interaction of 1,2,4-triazole and substances in the PEO-coating composition (Figure 7).

### 3.2. Analysis of the Electrochemical Properties

#### 3.2.1. EIS Data

**Figure 8:**
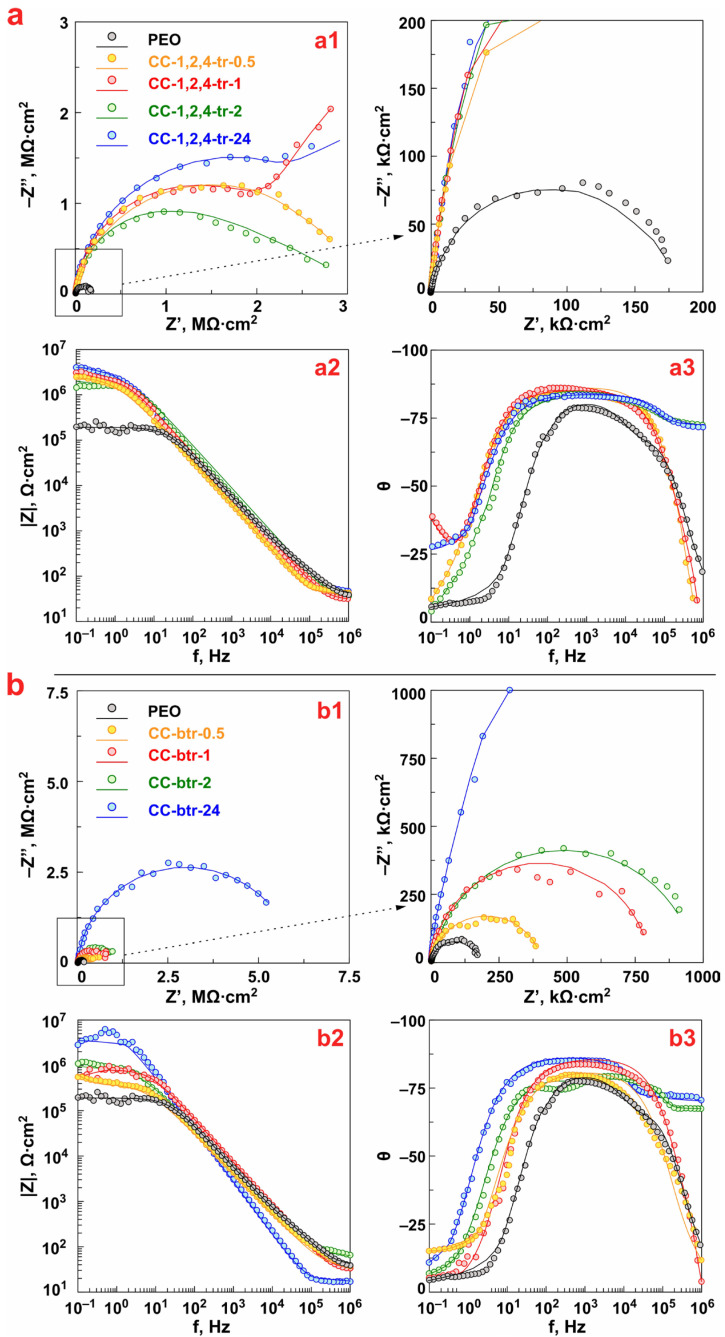
Nyquist (**a1**,**b1**) and Bode (**a2**,**b2**), (**a3**,**b3**) plots for samples with 1,2,4-triazole- (**a**) and benzotriazole-containing coatings (**b**) after 1 h exposure to 3.5 wt.% NaCl solution.

**Figure 9:**
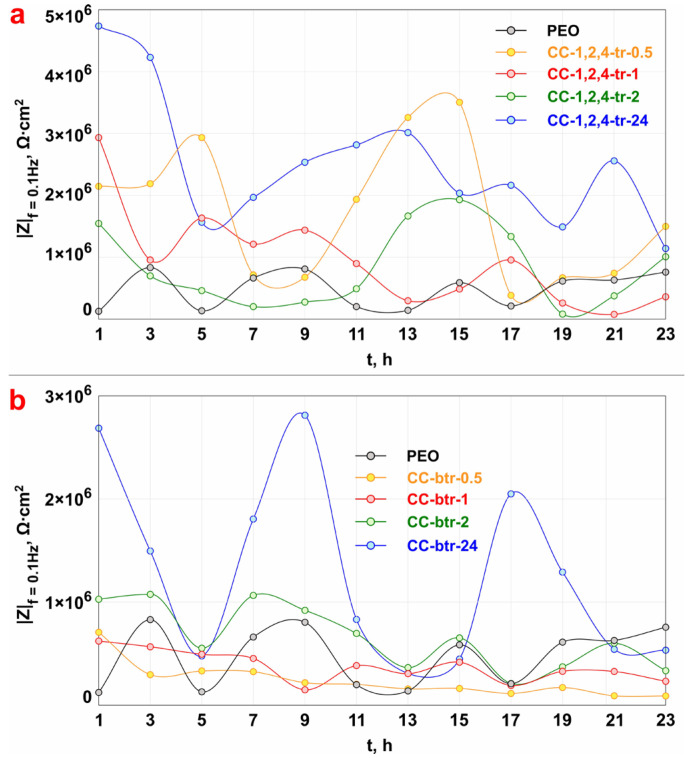
Evolution of |Z|*_f_*_=0.1Hz_ values for samples with 1,2,4-triazole- (**a**) and benzontriazole (**b**) containing coatings during 24 h of exposure to 3.5 wt.% NaCl solution.

Figure 8 shows the impedance spectra presented in the Nyquist and Bode plots, reflecting the corrosion behavior of the formed composite surface layers after 1 h of exposure to 3.5 wt.% NaCl solution. EIS spectra were acquired for the CC-1,2,4-tr-0.5 and CC-btr-0.5, CC-1,2,4-tr-1 and CC-btr-1, CC-1,2,4-tr-2 and CC-btr-2, CC-1,2,4-tr-24 and CC-btr-24 samples, assigned in Figure 8 and Figure 9 as 0.5 h, 1 h, 2 h, and 24 h, respectively. The spectrum for the sample with base PEO-layer is presented for comparison. 

The evolution of the impedance modulus measured at the lowest frequency *|Z|_f_*_=0.1Hz_ during the whole exposure time (24 h) of samples with different types of CC in sodium chloride solution is presented in Figure 9.

The study of the corrosion behavior of the samples, carried out by the EIS method, indicates an increase in the values of the impedance modulus *|Z|* over the entire frequency range after the increase in impregnation time of PEO-coating microtubes with corrosion inhibitors of the azole group up to 24 h (Figure 8). The best anticorrosion properties are observed for the CC-1,2,4-tr-24 sample, which is characterized by an increase in *|Z|_f_*_=0.1Hz_ values (*|Z|_f_*_=0.1Hz_ = 4.7 × 10^6^ Ω‧cm^2^) by 36 times in comparison with the base PEO-coating (*|Z|_f_*_=0.1Hz_ = 1.3 × 10^5^ Ω‧cm^2^) (Figure 8a, Table 3, Figure 9a). Impregnation of the PEO-coating with benzotriazole also has a positive effect on the level of anticorrosion protection of the obtained surface layers. The best protective properties among benzotriazole-containing CC were detected for the CC-btr-24 sample (Figure 8b, Table 3, Figure 9b). The value of impedance modulus measured at the lowest frequency for this type of coating reached 2.7 × 10^6^ Ω‧cm^2^, which is more than 20 times higher than the ones obtained for the base PEO-coating (*|Z|_f_*_=0.1Hz_ = 1.3 × 10^5^ Ω‧cm^2^). The increase in sample anticorrosion properties after the formation of azole-containing composite coating is also confirmed by a significant increase in the diameter of the semicircle on the Nyquist plot (Figure 8(a1,b1)).

Based on the analysis of the corrosion behavior data presented in Figure 9 for 1,2,4-triazole- and benzotriazole-containing samples it may be concluded, that 24 h is optimal and sufficient immersion time of the sample for the most complete loading of PEO-coating microtubes with a corrosion inhibitor. The impregnation during 0.5 h, 1 h, and 2 h of PEO-coated sample with the inhibitor-containing solution also results in an increase in the corrosion resistance as compared to the base PEO-layer. Probably it is related to the formation of azole-containing complexes with a substrate material and a PEO-layer. However, the analysis of the data presented in Figure 8 and Figure 9 allowed the work to reveal that the best anti-corrosion properties are provided after 24 h of immersion of the PEO-coated sample in the azole-containing solution. The CC-1,2,4-tr-24 and CC-btr-24 samples have higher values of the impedance modulus measured on the lowest frequency during 24 h of sample exposure to 3.5 wt.% solutions as compared to other types of coated specimens (Figure 9).

According to the results of the analysis of the data presented in Figure 9, the change of |Z|*_f_*_=0.1Hz_ values versus the loading time of the samples with formed surface layers in 3.5 wt.% NaCl for 24 h has an abrupt character. For the PEO-coating this behavior is explained due to possible partial degradation of the formed oxide layer and the formation of corrosion products. Alternately, increasing and decreasing values of the impedance modulus measured at the lowest frequency for composite inhibitor-containing coatings can characterize the self-healing properties of the protective layers. Such wave-like line trend of changing *|Z|_f_*_=0.1Hz_ was previously detected for the inhibitor-containing coatings formed on magnesium alloys and assigned as a self-healing effect [17].

**Figure 10:**
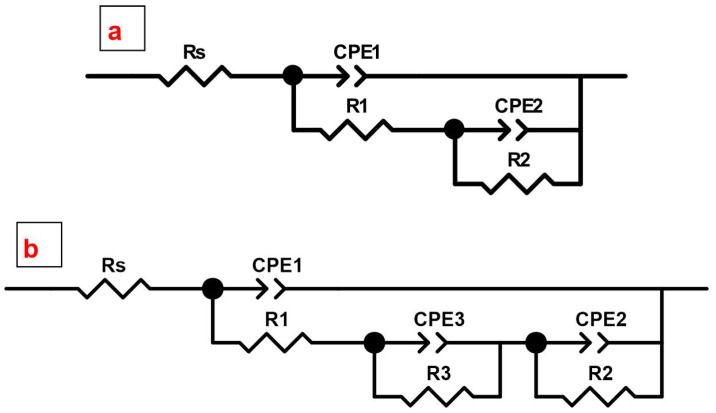
Equivalent electrical circuits (EEC) used for fitting the impedance spectra for PEO-, composite (**a**) and hybrid coatings (**b**).

Bode plots for PEO and all types of composite azole-containing coatings are characterized by the presence of two time constants (Figure 8(a2,b2)). Therefore, the fitting of the impedance spectra was performed using an equivalent electrical circuit (EEC) with two series-parallel *R-CPE* chains (Figure 10a, Table 3). The application of the constant phase element (*CPE*) instead of the *C* (ideal capacitance) is a result of the heterogeneity of the studied coatings. *R*_1_-*CPE*_1_ chain characterizes the resistive component (*R*_1_) of the outer (porous) part of the PEO-layer or coating treated with the inhibitor, as well as the geometric capacitance (*CPE*_1_) of the whole surface layer of formed coatings. The *R*_2_ and *CPE*_2_ elements describe resistive and capacitive components of an inner (non-porous) sublayer of the coating, including the inhibitor deposited at the bottom of the pores.

It has to be mentioned that samples with 1,2,4-triazole-containing composite coatings showed larger values of *R*_1_ + *R*_2_ and |Z|*_f_*_=0.1Hz_ than benzotriazole-containing ones obtained by corresponding immersion times (Table 3).

It can be assumed that this is due to the size of the molecules. The benzene part of the benzotriazole molecule can shield its reaction centers and cause steric hindrances leading to a decrease in the number of adsorbed molecules. While the smaller size of the 1,2,4-triazole molecule makes it possible to adsorb a larger number of molecules and contributes to an increase in the protective properties of composite coatings. 

**Figure 11:**
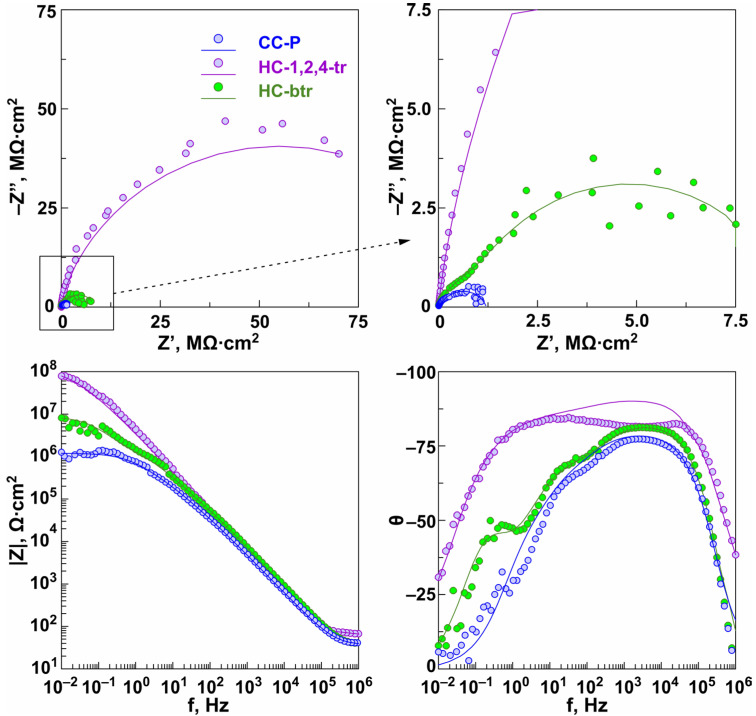
Nyquist and Bode plots for the HC-1,2,4-tr and HC-btr samples after 1 h exposure to 3.5 wt.% NaCl solution.

To improve the protective properties of the samples as well as to promote the prolongation of the self-healing effect, the specimens with composite inhibitor-containing coatings with the best anticorrosion properties (CC-1,2,tr-24, CC-btr-24) were treated with PVDF. These samples designated below as HC-1,2,4-tr and HC-btr, respectively, were studied by EIS. The curves for the CC-P sample are presented for comparison (Figure 11).

The impedance spectra presented in Figure 11 as Nyquist and Bode plots reflect the corrosion behavior of the formed surface layers of hybrid coatings after 1 h of exposure to 3.5 wt.% NaCl solution. The value of |Z|*_f_*_=0.1Hz_ for the HC-1,2,4-tr (*|Z|_f_*_=0.1Hz_ = 2.7 × 10^7^ Ω‧cm^2^) sample increases by more than two orders of magnitude in comparison with the base PEO-layer (*|Z|_f_*_=0.1Hz_ = 1.3 × 10^5^ Ω‧cm^2^) and by more than one order of magnitude in comparison with the CC-P sample (*|Z|_f_*_=0.1Hz_ = 1.0 × 10^6^ Ω‧cm^2^). For the HC-btr sample (*|Z|_f_*_=0.1Hz_ = 4.8 × 10^6^ Ω‧cm^2^) the increase in *|Z|_f_*_=0.1Hz_ was 37 and 5 times as compared to PEO-coated and CC-P samples. The application of the PVDF polymer enhances the protective properties of the sample with polymer-free coatings CC-1,2,4-tr-24 (*|Z|_f_*_=0.1Hz_ = 4.7 × 10^6^ Ω‧cm^2^) and CC-btr-24 (*|Z|_f_*_=0.1Hz_ = 2.7 × 10^6^ Ω‧cm^2^) by 6 and 2 times, respectively (Figure 11, Table 3 and Table 4). The HC-1,2,4-tr sample showed the highest anticorrosion performance. The Bode plots for the CC-P, HC-1,2,4-tr, and HC-btr samples are characterized by the presence of three time constants. Therefore, the fitting of the obtained spectra was implemented using an equivalent electrical circuit with three series-parallel *R-CPE* chains (Figure 10b, Table 4). For this type of hybrid coating, *R*_1_*-CPE*_1_ chain characterizes the resistance of the upper layer, formed by a polymer component, deposited on the top and in the pores of the formed coating and promoted the partial sealing the microcontainers with an inhibitor, and also takes into account the capacitive component of the entire composite coating. *R*_2_-*CPE*_2_ chain represents the inner (non-porous) sublayer of the PEO-coating and *R*_3_-*CPE*_3_ is used to describe the porous part of the PEO-layer impregnated with benzotriazole or 1,2,4-triazole. The change of the parameter *R*_1_, *R*_2_, and *R*_3_ (resistance of the upper layer, inner layer, and porous part of the hybrid coating, respectively) also indicates the higher corrosion resistance of the sample with hybrid 1,2,4-triazole-containing coating as compared to one for the HC-btr specimen.

The protective properties of the formed surface layers are comparable with the results obtained by various scientific groups dealing with the formation of the base and modified PEO-coatings on Al alloys. According to the analyzed literature data the values of the impedance modulus measured at the lowest frequency (*|Z|_f_*_=0.1Hz_ or *|Z|_f_*_=0_._01Hz_) as well as the polarization resistance of the coatings studied in [4,5,18,19,20,23,25] changed in the range from 10^3^ Ω‧cm^2^ up to 10^7^ Ω‧cm^2^. These results show the high corrosion resistance of the hybrid coatings formed and studied in this work. Moreover, due to coating impregnation with the inhibitor, these layers can provide active corrosion protection of the treated Al alloy. 

#### 3.2.2. SVET/SIET Data

**Figure 12:**
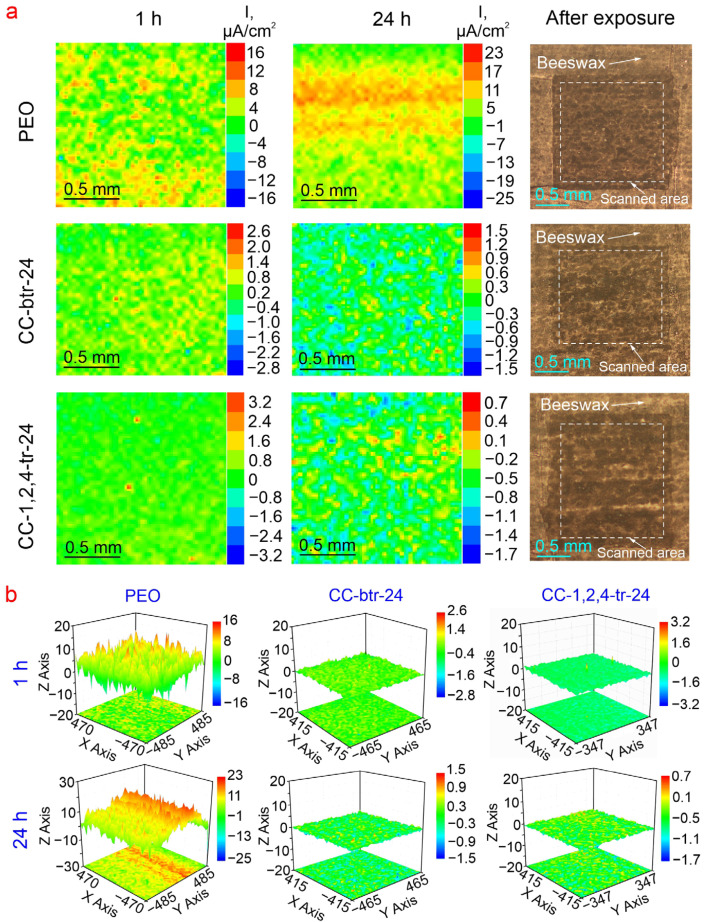
SVET maps (color scale depicts the local current density in µA cm^2^) and optical images of scanned areas of samples with different types of coatings (**a**) and comparison of the intensity of samples’ local electrochemical activity presented in 3D maps in PEO axis scale (**b**).

**Figure 13:**
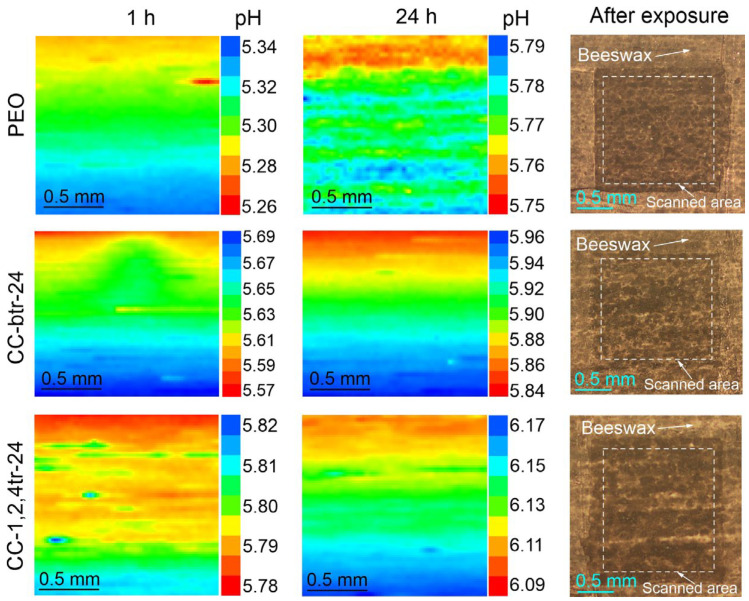
pH distribution (SIET) maps and optical images of scanned areas of samples with different types of coatings.

Figure 12a and Figure 13 present maps of local current density and pH distribution over the scanned area of studied samples (with PEO-layer, CC-btr-24, and CC-1,2,4-tr-24) after 1 h and 24 h of exposure to 0.05 M NaCl solution. To study the effect of the inhibitor on the corrosion process a comparison of SVET/SIET results was made only between CC and PEO-coated samples. The scanned area is highlighted by a frame. The development of a corrosion process for the PEO-coated sample is detected during the first hour of the immersion in a corrosive media (Figure 12a and Figure 13), according to SVET and SIET data. Formation of anodic zones (yellow and red areas of the presented maps) with higher values of the anodic current and lower values of pH are associated with the reactions of aluminum dissolution and hydrolysis, which are characteristic of the corrosion of this material [63]). Further exposure is characterized by the increase in both local current densities and pH values (Figure 12 and Figure 13 (24 h)). This is explained by the possible partial degradation of a protective layer with the following dissolution of the magnesium, which is presented in the composition of the substrate. Since magnesium is a very active metal and its corrosion process is accompanied by high alkalization [64,65], its presence in the alloy composition makes a significant impact on the observed corrosion pattern with the increase of the exposure time to a corrosive media [66,67] (Figure 13). Impregnation of the micro-containers of the PEO-layer with corrosion inhibitors 1,2,4-triazole and benzotriazole leads to a significant decrease in the level of electrochemical activity of studied samples (Figure 12 and Figure 13). The stabilization of corrosion behavior expressed in lower values of the local current density for CC-btr-24 and CC-1,2,4-tr-24 samples as well as the higher local pH (as compared to ones for the sample with base PEO-layer) after 24 h of immersion in sodium chloride solution represents the manifestation of a self-healing effect of the formed composite coatings. The less intense decrease in the pH value, as compared to the specimen with an inhibitor-free layer, and approaching the neutral initial value of the pH indicates the low intensity of the corrosion process of the sample with the composite coating (Figure 12 and Figure 13). It should be noted, that for a sample with 1,2,4-triazole-containing composite coating after 24 h of exposure, the pH values shifted closer to the ones characteristic of 0.05 M NaCl solution (ca. pH = 7.0). Figure 12b depicts the 3D maps of the current density distribution over the specimen surface. Maps are shown at the same scale for better comparison of the protective properties of the coated samples. These results clearly demonstrate the higher corrosion stability of the CC-btr-24 and CC-1,2,4-tr-24 specimens as compared to one with the base PEO-layer. Based on a complex analysis of the presented data, it can be concluded that CC-1,2,4-tr-24 samples demonstrate the best anti-corrosion properties among all of the studied surface layers. The presented results are in agreement with the data obtained by electrochemical impedance spectroscopy.

### 3.3. Adhesion Analysis

**Figure 14:**
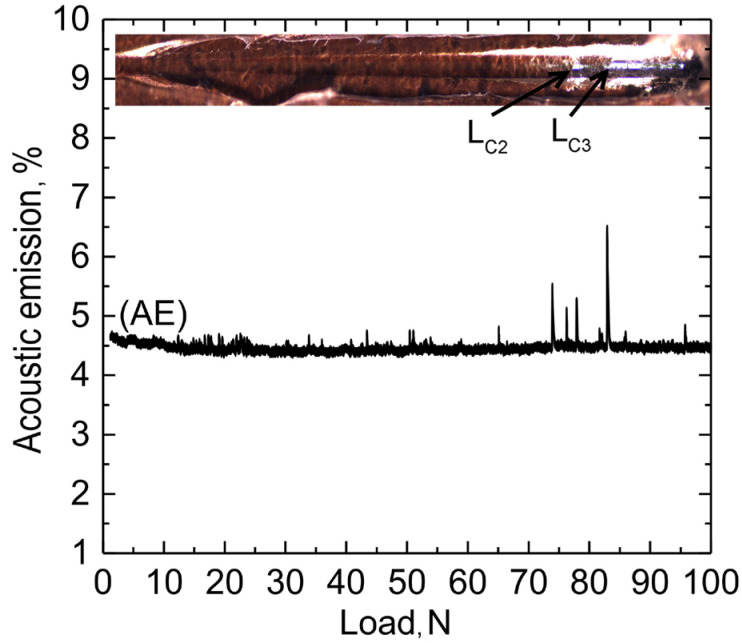
The photograph of the HC-1,2,4-tr sample after the scratch test and the dependence of acoustic emission (AE) on the load on the indenter at a speed of 12 N min^−1^.

According to the photo of the scratch and acoustic emission (AE) data, at a speed of 12 N min^−1^, the indenter scratched the HC-1,2,4-tr specimen at a load L_C3_ = 83 ± 3 N (L_C3_ is a load at which the coating is abraded to the metal substrate, Figure 14). The first evidence of the destruction of the protective layer is observed at L_C2_ = 77 ± 3 N. These results indicate the high mechanical characteristics and adhesion of the inhibitor-containing PEO-coating treated with PVDF.

### 3.4. Corrosion Inhibition Mechanism

**Figure 15:**
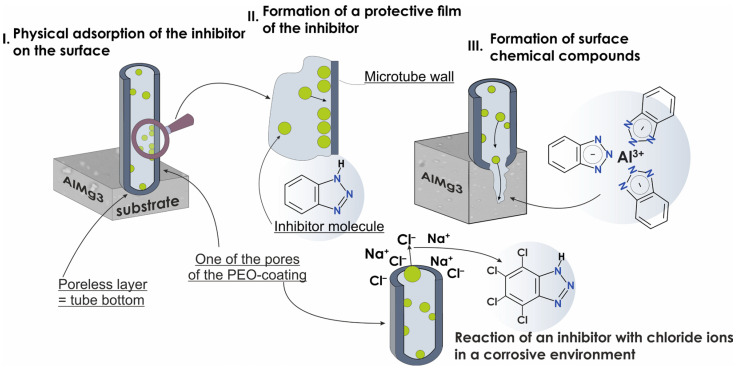
The mechanism of the protection against corrosion of the aluminum alloy sample with azole-containing PEO-layer in the presence of chlorine ions. Benzotriazole was used as an example of the action of the corrosion inhibitor.

The complex mechanism of corrosion inhibition is described in this work. The stages of protecting the AlMg3 aluminum alloy against corrosion in the aggressive chloride-containing environment are presented in the scheme in Figure 15. Benzotriazole was used as an example of a corrosion inhibitor impregnated into the pores of a PEO-coating. It is assumed that the inhibition of aluminum corrosion processes by azoles in the presence of a PEO-coating reflects the following phenomena:

1. Active physical adsorption of triazoles on the surface of the PEO-coating due to the high affinity of aluminum and magnesium (which is the main alloying element in the AlMg3 alloy) to nitrogen (Figure 15, stage I). At the first stage, aggressive chloride ions can be captured by the corrosion inhibitor to form the compound 4,5,6,7-tetrachloro-1H-1,2,3-benzotriazole (as presented in Figure 15) [68].
2. Formation of the protective film of a colloid-dispersed inhibitor (Figure 15, stage II).
3. Formation of surface chemical compounds. Triazoles can act as ligands for aluminum (at the sites of damaged or degraded part of the PEO-layer and zones of the bare alloy) with subsequent formation of an organometallic compound, as shown in Figure 15, stage III.

It should be noted, that in the process of chemisorption realized at the third stage, the corrosion inhibitor can bind not only to the metal surface but also to the PEO-layer with the formation of complex compounds.

## 4. Conclusions

The method of improving the protective properties of AlMg3 aluminum alloy by the formation of hybrid inhibitor-containing coatings based on the oxide PEO-layer with a self-organized microtubular structure was suggested and optimized. The following results were obtained:

- The optimal way of the impregnation of the PEO-coating matrix with organic corrosion inhibitors–benzotriazole and 1,2,4-triazole was selected (24 h immersion in 0.05 M solution). The presence of the corrosion inhibitor in the PEO-coating microtubes was confirmed and their distribution over the surface layer was established;
- A controlled release of the inhibitory agents from the pores-microcontainers of PEO-coating was ensured by processing the obtained composite coatings with a polymer material–polyvinylidene fluoride (PVDF) using the spray-coating method. This method allows the prolongation of the action of active corrosion protection of the substrate material. The HC-1,2,4-tr sample is characterized by the best corrosion resistance (*|Z|_f_*_=0.1Hz_ = 2.7 × 10^7^ Ω‧cm^2^). The protective properties of the surface layers are comparable with the results obtained by various scientific groups dealing with the formation of the base and modified PEO-coatings on Al alloys. Moreover, our hybrid layers have high adhesion to the substrate and can provide active corrosion protection of the Al alloy.
- It was revealed, that the impregnation of the PEO-coating’s microtubes with benzotriazole and 1,2,4-triazole leads to a significant decrease in local electrochemical activity evaluated at the microscale. Lower values of the local current density and higher pH values (as compared to ones registered for the sample with inhibitor-free PEO-layer) in the solution near the studied surface were detected;
- Based on the results of the complex analysis of the protective properties of the formed surface layers, the corrosion inhibition mechanism of hybrid inhibitor- and polymer-containing coating was suggested. The results of this work are beneficial for better understanding the degradation behavior of aluminum and its alloys and moving forward in developing an effective method for anticorrosion protection of promising constructional material.

## Figures and Tables

**Table 1:** Binding energy (eV) and elemental composition (at. % in parentheses) of the surface of CC-1,2,4-tr-24 sample.

Element	Chemical State	Studied Surface	
		Before Etching	After Etching
F (1s)	CF_x_	689.3 (0.5)	687.8 (0.5)
	MF *	685.8 (1.1)	686.1 (1.2)
O (1s)	CO_x_	533.1 (20.1)	533.0 (21.0)
	Al_2_O_3_	531.7 (17.9)	531.6 (19.4)
N (1s)	-NH-	400.4 (1.1)	400.7 (0.8)
	=N-	399.1 (0.6)	398.5 (0.6)
C (1s)	O-C = O;	288.6 (3.7)	288.9 (2.7)
	C-O-C; C-N	286.4 (9.3)	286.4 (5.1)
	C-C; C-H	285.0 (24.0)	285.0 (12.5)
	MC *	-	283.0 (6.4)
Si (2p)	SiO	102.0 (1.3)	102.0 (1.5)
	SiC	100.3 (2.6)	100.5 (3.1)
Al (2p)	Al^3+^	74.4 (17.0)	75.0 (24.1)
Mg (2s)	Mg^2+^	90.0 (0.8)	91.4 (1.1)

* Abbreviations MF and MC indicate a metal fluoride and metal carbide, respectively.

**Table 2:** Binding energy (eV) and elemental composition (at. % in parentheses) of the surface of CC-btr-24 sample.

Element	Chemical State	Studied Surface	
		Before Etching	After Etching
F (1s)	CF_x_	689.0 (0.4)	687.8 (0.4)
	MF *	685.4 (1.2)	686.3 (1.2)
O (1s)	CO_x_	532.7 (20.9)	533.0 (23.4)
	Al_2_O_3_	531.4 (15.1)	531.7 (19.5)
N (1s)	-NH-	400.6 (2.4)	400.4 (1.0)
	=N-	399.3 (0.9)	398.3 (0.8)
C (1s)	O-C = O	288.5 (5.7)	288.4 (0.8)
	C-O-C; C-N	286.3 (9.7)	286.3 (5.6)
	C-C; C-H	285.0 (25.2)	285.0 (10.8)
	MC *	-	283.2 (3.5)
Si (2p)	SiO	101.8 (1.0)	101.7 (1.8)
	SiC	99.9 (2.2)	100.0 (3.4)
Al (2p)	Al^3+^	74.8 (14.8)	75.0 (27.3)
Mg (2s)	Mg^2+^	89.0 (0.5)	89.5 (0.5)

* Abbreviations MF and MC indicate a metal fluoride and metal carbide, respectively.

**Table 3:** Calculated parameters of EEC elements for samples of AlMg3 aluminum alloy with various types of CC and PEO-layer.

Sample	*CPE*_1_		*R*_1_, Ω∙cm^2^	*CPE*_2_		*R*_2_, Ω∙cm^2^	|*Z*|*_f_*_=0.1Hz_, Ω∙cm^2^
	*Q*_1_, S∙cm^−2^∙c^n^	*n*		*Q*_2_, S∙cm^−2^∙c^n^	*n*		
PEO	4.8 × 10^−8^	0.91	1.1 × 10^3^	1.6 × 10^−8^	0.93	1.7 × 10^5^	1.3 × 10^5^
CC-1,2,4-tr-0.5	4.2 × 10^−8^	0.96	8.8 × 10^5^	7.9 × 10^−8^	0.63	2.3 × 10^6^	2.1 × 10^6^
CC-1,2,4-tr-1	4.6 × 10^−8^	0.95	2.5 × 10^6^	5.8 × 10^−7^	0.97	6.3 × 10^6^	2.9 × 10^6^
CC-1,2,4-tr-2	4.1 × 10^−8^	0.94	2.7 × 10^6^	7.0 × 10^−7^	0.98	6.2 × 10^6^	1.5 × 10^6^
CC-1,2,4-tr-24	4.1 × 10^−8^	0.94	2.9 × 10^6^	1.5 × 10^−7^	0.31	8.0 × 10^6^	4.7 × 10^6^
CC-btr-0.5	9.4 × 10^−8^	0.90	1.9 × 10^5^	1.2 × 10^−7^	0.57	2.0 × 10^5^	7.1 × 10^5^
CC-btr-1	1.1 × 10^−7^	0.88	2.1 × 10^4^	1.0 × 10^−8^	0.87	4.4 × 10^5^	5.7 × 10^5^
CC-btr-2	7.3 × 10^−8^	0.89	3.6 × 10^5^	2.4 × 10^−8^	0.94	6.3 × 10^5^	1.1 × 10^6^
CC-btr-24	2.8 × 10^−8^	0.95	4.0 × 10^6^	8.7 × 10^−8^	0.26	2.1 × 10^6^	2.7 × 10^6^

**Table 4:** The calculated parameters of EEC elements for samples of AlMg3 aluminum alloy with various types of HC formed on the basis of a PEO-layer.

Sample	*CPE*_1_		*R*_1_, Ω∙cm^2^	*CPE*_2_		*R*_2_, Ω∙cm^2^	*CPE*_3_		*R_3_*, Ω∙cm^2^	|*Z*|*_f_*_=0_._1Hz,_ Ω∙cm^2^
	*Q*_1_, S∙cm^−2^∙c^n^	*n*		*Q*_2_, S∙cm^−2^∙c^n^	*n*		*Q*_3_, S∙cm^−2^∙c^n^	*n*		
HC-1,2,4-tr	2.9 × 10^−8^	0.97	5.1 × 10^5^	4.3 × 10^−8^	0.71	7.9 × 10^7^	3.8 × 10^−8^	0.62	3.4 × 10^7^	2.7 × 10^7^
HC-btr	3.8 × 10^−8^	0.94	1.2 × 10^5^	2.8 × 10^−7^	0.83	7.5 × 10^6^	9.3 × 10^−8^	0.70	1.2 × 10^6^	4.8 × 10^6^
CC-P	7.7 × 10^−8^	0.89	1.0 × 10^5^	1.8 × 10^−7^	0.68	2.7 × 10^5^	2.8 × 10^−7^	0.74	8.1 × 10^5^	1.0 × 10^6^

## Data Availability

Not applicable.
